# National Differences in Remission of Type 2 Diabetes Mellitus After Roux-en-Y Gastric Bypass Surgery-Subgroup Analysis of 2-Year Results of the Diabetes Surgery Study Comparing Taiwanese with Americans with Mild Obesity (BMI 30–35 kg/m^2^)

**DOI:** 10.1007/s11695-016-2433-4

**Published:** 2016-10-25

**Authors:** Keong Chong, Sayeed Ikramuddin, Wei-Jei Lee, Charles J. Billington, John P. Bantle, Qi Wang, Avis J. Thomas, John E. Connett, Daniel B. Leslie, William B. Inabnet, Robert W. Jeffery, Michael G. Sarr, Michael D. Jensen, Adrian Vella, Leaque Ahmed, Kumar Belani, Joyce L. Schone, Amy E. Olofson, Heather A. Bainbridge, Patricia S. Laqua, Judith Korner, Lee-Ming Chuang

**Affiliations:** 1 Department of Medicine, Min-Sheng General Hospital, Taoyuan City, Taiwan; 2 Department of Surgery, University of Minnesota, Minneapolis, MN USA; 3 Department of Surgery, Min-Sheng General Hospital, Taoyuan City, Taiwan; 4 Department of Medicine, Division of Endocrinology and Diabetes, University of Minnesota, Minneapolis, MN USA; 5 Division of Biostatistics, University of Minnesota, Minneapolis, MN USA; 6 Department of Surgery, Mount Sinai Beth Israel, New York, NY USA; 7 Division of Epidemiology and Community Health, School of Public Health, University of Minnesota, Minneapolis, MN USA; 8 Department of Gastroenterologic and General Surgery, Mayo Clinic, Rochester, MN USA; 9 Department of Medicine, Division of Endocrinology and Diabetes, Mayo Clinic, Rochester, MN USA; 10 Department of Surgery, Harlem Hospital Center, New York, NY USA; 11 Department of Anesthesiology, University of Minnesota, Minneapolis, MN USA; 12 Nutrition Services, Fairview Health System, Minneapolis, MN USA; 13 Department of Bariatric Surgery, Temple University Hospital, Philadelphia, PA USA; 14 Department of Medicine, Division of Endocrinology, Columbia University Medical Center, New York, NY USA; 15 Department of Medicine, National Taiwan University Hospital, Taipei, Taiwan; 16 Department of Internal Medicine, National Taiwan University Hospital, No. 7, Chung-Shan South Road, Taipei, 100 Taiwan

**Keywords:** Diabetes remission, Type 2 diabetes mellitus, Roux-en-Y gastric bypass, National differences, Ethnic differences, Taiwanese

## Abstract

**Background:**

The purpose of this study is to compare effects of different nations on Roux-en-Y gastric bypass (RYGB) vs. intensive medical management (IMM) in achieving remission of type 2 diabetes mellitus (T2DM).

**Materials and Methods:**

Between April 2008 and December 2011, this randomized, controlled clinical trial was conducted at four teaching hospitals in the United States and Taiwan involving 71 participants with mild obesity (BMI 30–35 kg/m^2^). Thirty-six of 71 participants were randomly assigned to the RYGB group, and the others were in IMM group. Partial or complete remission of T2DM was defined as blood HbA1c < 6.5 % (48 mmol/mol) or <6 % (42 mmol/mol) without any antihyperglycemic medication for at least 1-year duration, respectively.

**Results:**

At baseline, Taiwanese participants had a lower BMI, younger age, and shorter duration of T2DM than American participants. At 24 months, weight loss was greater in the RYGB group in both populations than in the IMM group. No IMM participant of either population had partial or complete remission of T2DM. In the RYGB group, a substantial proportion of the subjects achieved complete or partial remission (57 % in Taiwanese and 27 % in American participants, *P* = 0.08). Logistic regression revealed stimulated C-peptide (Odds ratio 2.22, *P* = 0.02) but not nationality as a significant predictor of diabetes remission.

**Conclusion:**

Adding RYGB to lifestyle and medical management was associated with a greater likelihood of remission of T2DM in both Taiwanese and American subjects with mild obesity with type 2 diabetes. Residual beta-cell function at baseline appears to be the major factor predicting remission of T2DM.

Trial registry number: clinicaltrials.gov Identifier: NCT00641251

## Introduction

Type 2 diabetes mellitus (T2DM) is a major chronic debilitating disease worldwide, affecting more than 387 million people, and that number is expected to 592 million by the year 2035 [1]. The development of T2DM is strongly associated with central obesity or the accumulation of intra-abdominal fat. T2DM is a particular problem in Asia because of a tendency to develop T2DM at a lower BMI [2]. The prevalence has recently increased 3–6 fold within 20–25 years in Asia [3]. In Taiwan, T2DM in adults has more than doubled from 4.6 to 9.3 % of the population in the past decade [4]. Unlike in the West where the older population is the most affected, the burden of diabetes in Asian countries is disproportionately high in young to middle-age adults [2, 3].

Controlling this chronic and debilitating disease is a very important health issue in Asia. Medications to improve glycemia and control cardiovascular risk are important, but up to 90 % of patients with T2DM do not achieve treatment goals designed to decrease the long-term risk of complications [5]. In the 2007–2010 diabetic NHANES population, 52.5 % of people with diabetes achieved a blood HbA1c < 7.0 % (<53 mmol/mol), 51.1 % achieved BP < 130/80 mmHg, 56.2 % achieved LDL < 100 mg/dL (2.586 mmol/L), and 18.8 % achieved all three as a composite goal [6]. In a similar 2011 Taiwan national survey after a policy of diabetes awareness, 34.5 % of people with diabetes achieved HbA1c < 7 % (<53 mmol/mol), 37.7 % achieved a blood pressure target <130/80 mmHg, 55.7 % achieved total cholesterol <160 mg/dl (4.14 mmol/L) or LDL cholesterol lower than 100 mg/dl (2.59 mmol/L), and 8.6 % achieved all three [7].

Bariatric surgery has been advocated to improve the disordered metabolism present in T2DM. Evidence favoring improvement in the outcomes of T2DM after bariatric surgery is provided by observational studies [8, 9] and early reports from randomized trials [10, 11] in both Taiwanese and American cohorts. A meta-analysis of ethnic differences in weight loss and remission of T2DM after bariatric surgery had been published [12], but no comparison between Asian and American has been reported. The Diabetes Surgery Study is the first randomized, controlled trial to enroll both Taiwanese and American participants.

This subgroup analysis aimed to compare the impact of RYGB with lifestyle modification with IMM alone to achieve remission of T2DM in Asians compared to a US cohort with BMI 30–35 kg/m^2^.

## Methods

### Study Design and Participants

The DSS study design and participants have been previously reported [13, 14]. The study was conducted at four sites: the University of Minnesota, Columbia University Medical Center in New York, the Mayo Clinic in Rochester, and two academic clinics in Taiwan (National Taiwan University Hospital and Min Sheng General Hospital, together called Taiwan). Institutional Review Board approval and written informed consent from each patient was obtained at all sites.

Inclusion and exclusion criteria have been also reported previously [13, 14]. Key inclusion criteria included blood levels of HbA1c of 8.0 % (64 mmol/mol) or greater despite at least 6 months of care by a physician for T2DM, BMI 30.0–35 kg/m^2^, and willingness and ability to accept randomization and follow the full treatment protocol. Exclusion criteria included the conditions that would contraindicate surgery, such as serious cardiovascular disease, previous gastrointestinal surgery, psychologic concerns, or history of malignancy.

Patients were randomized within sites to either intensive medical management (IMM) or lifestyle and medical management plus a RYGB, using a random, permuted block design. Clinical center personnel had access to data on their individual patients but were blinded to data on other patients and to aggregated data until all two-year follow-up data had been obtained.

### Procedures

The procedures of DSS have been previously reported [13, 14]. The lifestyle intervention was modeled on recent, successful clinical trials, particularly the Diabetes Prevention Program (DPP) [15] and the Look AHEAD protocol [16]. Visits with an endocrinologist occurred monthly for 6 months and then quarterly (or monthly if not at goal) for the next 6 months, then quarterly through the second year. Medications for glycemic control were added or reintroduced in the following order: metformin, a glucagon-like peptide-1 receptor agonist or a dipeptidyl peptidase-4 inhibitor, sulfonylurea or pioglitazone, and insulin.

The technique of RYGB was standardized across all sites and performed with construction of a 20-mL lesser curvature gastric pouch a 100-cm biliopancreatic limb. All surgeons committed to following this protocol which was reviewed at an onsite meeting. The technical skill of each surgeon was established by personal observation of the principal surgeon. The study surgeons performed all postoperative surgical interventions.

### Outcomes

Partial remission of T2DM was defined as HbA1c < 6.5 % (48 mmol/mol), and complete remission as HbA1c < 6.0 % (42 mmol/mol), at every sampling from months 12 to 24 with no medicines for hyperglycemia in the RYGB group. These definitions are in line with the recommendations of a consensus statement by the American Diabetes Association [17]. We obtained data at baseline, medical visits, and lifestyle intervention visits. For each group, baseline measurements were taken at the time of randomization. The data included measurements of height, weight, blood pressure, waist circumference, medication used, and adverse events. Laboratory measurements included blood levels of HbA1c, fasting lipid profile, complete blood cell count, electrolytes, hepatic panel, ferritin, vitamin B1, vitamin B12, vitamin D, parathyroid hormone, calcium, fasting blood glucose and C-peptide levels, 90-min post-meal glucose and C-peptide levels, and urine microalbumin to creatinine ratios.

### Statistical Analysis

Statistical analyses were performed in SAS 9.3 (SAS Institute, Cary NC). All analyses were done on an intention to treat basis. Multiple imputations were conducted to address the issue of missing data using PROC MI in SAS. Forty imputations were done. Data from baseline, 12 and 24 months were included in the model. Information on crossover was also included.

Data are presented as mean (SD) for continuous variables and *N* (%) for categorical variables. Dichotomous data were analyzed using logistic regressions stratified by site. Continuous data were analyzed using linear regressions adjusted for site. Regressions were done separately for each imputation and then summarized using PROC MIANALYZE.

Potential predictors of remission were examined individually using logistic regressions, and those variables with *P* < 0.10 were included in the multivariate models. Odds ratios and their 95 % confidence intervals are presented.

## Results

Between April 2008 and December 2011, 120 patients were randomized either to the IMM or RYGB groups of whom 71 patients had mild obesity (BMI 30–35 kg/m^2^); 35 participants (19 Americans and 16 Taiwanese) were enrolled in IMM group, and 36 participants (22 Americans and 14 Taiwanese) in RYGB group.

Baseline characteristics of two populations with mild obesity are shown in Table 1. BMI (31.9 ± 1.7 vs. 32.9 ± 1.5 kg/m^2^, *P* = 0.016) and waist circumference (102.7 ± 6.4 vs. 112.2 ± 7.4 cm, *P* < 0.001) in Taiwanese participants were significantly less than in American participants. Taiwanese participants also tended to be younger (45.7 ± 7.9 years vs. 50.7 ± 8.9 years, *P* = 0.014). The duration of T2DM in Taiwanese was less (6.1 ± 3.6 vs. 10.4 ± 6.5 years, *P* = 0.0006), while fewer Taiwanese took insulin (33 % vs. 59 %, *P* = 0.036). More American participants took blood pressure medications (76 % vs. 43 %, 0.006), but no significant difference in baseline systolic blood pressure were noted. Fasting serum levels of C-peptide and 90-min postprandial C-peptide (stimulated C-peptide) did not differ in the two populations.

**Table 1:** Baseline characteristics

	Taiwan (*N* = 30)	US (*N* = 41)	*P* value
Age	45.7 (7.9)	50.7 (8.9)	0.014
Female	22 (73 %)	24 (59 %)	0.20
Race/Ethnicity			
Non-Hispanic White	0	31 (76 %)	
Asian	30 (100 %)	1 (2 %)	–
Non-Hispanic Black	0	4 (10 %)	
Hispanic	0	3 (7 %)	
Native American	0	0 (0 %)	
Other	0	2 (5 %)	
BMI (kg/m^2^)	31.9 (1.7)	32.9 (1.5)	0.016
Height (cm)	161.5 (6.4)	170.4 (10.3)	<.0001
Weight (kg)	83.4 (8.3)	95.8 (12.5)	<.0001
Waist circumference (cm)	102.7 (6.4)	112.2 (7.4)	<.0001
SBP (mmHg)	128.0 (14.7)	127.7 (13.1)	0.93
DBP (mmHg)	81.8 (9.4)	74.3 (10.1)	0.002
Years since diagnosis of T2DM	6.1 (3.6)	10.4 (6.5)	0.0006
Blood HbA1c (%)	9.56 (1.14)	9.76 (1.25)	0.49
LDL cholesterol (mmol/L)	2.92 (0.92)	2.59 (1.19)	0.19
HDL cholesterol (mmol/L)	1.08 (0.22)	1.10 (0.30)	0.68
Triglycerides (mmol/L)	2.46 (2.08)	3.24(5.34)	0.40
Total cholesterol (mmol/L)	4.75 (0.98)	4.85 (1.35)	0.70
Creatinine (μmol/L)	65.1 (14.4)	69.00 (18.64)	0.33
Fasting C-peptide (nmol/L)	1.05 (0.66)	0.90 (0.45)	0.28
Stimulated C-peptide (nmol/L)	1.70 (1.00)	1.35 (0.61)	0.093
Fasting glucose (mmol/L)	10.88 (3.04)	13.22 (4.49)	0.011
Taking insulin	10 (33 %)	24 (59 %)	0.036
Taking other glycemic medicines	28 (93 %)	37 (90 %)	1
Taking dyslipidemia medicines	16 (53 %)	29 (71 %)	0.13
Taking blood pressure medicines	13 (43 %)	31 (76 %)	0.006
Number of medications for control of glycemia, dyslipidemia, and blood pressure	3.4 (1.3)	4.2 (1.4)	0.015

*US* United States, *BMI* body mass index, *SBP* systolic blood pressure, *DBP* diastolic blood pressure, *HbA1c* glycated hemoglobin, *LDL cholesterol* low density lipoprotein cholesterol, *HDL cholesterol* high density lipoprotein cholesterol

Table 2 shows the outcomes of two populations in the IMM and RYGB group at 24 months. In the IMM group, no significant difference in achieving treatment targets and major metabolic markers were found in these two populations. There were fewer Taiwanese taking insulin and blood pressure medications. In the RYGB group, patients lost more weight than in the IMM group, but in the RYGB group, there were no significant difference in percent weight change (−21.2 ± 6.1 vs. −24.52 ± 10.7 %, *P* = 0.45) was noted between these two populations. Taiwanese participants had greater reduction in SBP and DBP but no significant different in achieving the targets of SBP < 130 mmHg (86 % vs. 90 %, *P* = 0.84). Both populations had similar effect on glucose reduction and achieving HbA1c target (86 % vs. 67 %, *P* = 0.20).

**Table 2:** Key outcomes at 24 months

	Lifestyle and medical management			Roux-en-Y gastric bypass		
	Taiwan (*N* = 16)	US (*N* = 19)	*P* value	Taiwan (*N* = 14)	US (*N* = 22)	*P* value
Meets triple endpoint	3 (19 %)	1 (7 %)	0.25	5 (36 %)	11 (52 %)	0.40
HbA1c < 7.0 % (53 mmol/mol)	3 (19 %)	3 (20 %)	0.99	12 (86 %)	14 (67 %)	0.20
LDL cholesterol <100 mg/dL	13 (81 %)	10 (67 %)	0.17	8 (57 %)	17 (81 %)	0.20
SBP < 130 mmHg	13 (81 %)	10 (67 %)	0.21	12 (86 %)	19 (90 %)	0.84
Change in weight (kg)	−4.2 (4.5)	−8.1 (7.9)	0.36	−18.4 (5.9)	−24.2 (11.2)	0.13
Percent weight change	−5.1 (5.5)	−8.8 (8.2)	0.48	−21.2 (6.1)	−24.5 (10.7)	0.45
Change in BMI	−1.9 (1.9)	−2.8 (2.5)	0.47	−6.9 (2.1)	−8.1 (3.6)	0.34
Change in waist circumference (cm)	−4.8 (5.1)	−8.2 (9.1)	0.21	−20.8 (9.7)	−22.2 (9.7)	0.68
Change in fasting glucose (mmol/L)	−1.04 (3.49)	−2.35 (4.42)	0.11	−5.30 (2.64)	−6.50 (5.22)	0.32
Change in HbA1c (%)	−0.59 (1.52)	−1.41 (1.56)	0.19	−3.24 (1.05)	−2.83 (1.58)	0.49
Change in LDL-C (mmol/L)	−0.38 (1.19)	−0.39 (1.27)	0.87	−0.87 (1.16)	−0.30 (0.82)	0.09
Change in HDL-C (mmol/L)	−0.02 (0.22)	0.02 (0.23)	0.75	0.24 (0.21)	0.24 (0.45)	0.97
Change in triglycerides (mmol/L)	0.21 (3.20)	−0.13 (2.17)	0.98	−0.92 (0.65)	−2.30 (6.92)	0.48
Change in total cholesterol (mmol/L)	−0.51 (1.47)	−0.26 (1.51)	0.57	−0.60(1.37)	−0.68 (1.10)	0.82
Change in creatinine (μmol/L)	0 (9.7)	6.1 (20.5)	0.30	−7.58 (7.55)	−0.55 (20.0)	0.22
Change in fasting C-peptide (nmol/L)	−0.13 (0.36)	−0.11 (0.42)	0.57	−0.44 (0.98)	−0.22 (0.34)	0.38
Change in post-meal C-peptide (nmol/L)	−0.08 (0.72)	0.01 (0.65)	0.67	−0.27 (1.31)	0.11 (0.70)	0.35
Change in SBP	−9.1 (12.4)	−2.8 (18.6)	0.19	−16.1 (14.1)	−5.8 (14.4)	0.03
Change in DBP	−4.1 (7.9)	-0.5 (10.1)	0.54	−13.2 (6.7)	−5.8 (10.1)	0.02
Taking Insulin	3 (19 %)	9 (60 %)	0.03	0 (0 %)	4 (19 %)	0.13
Taking other glycemic medicines	15 (94 %)	14 (93 %)	0.42	4 (29 %)	11 (52 %)	0.18
Taking dyslipidemia medicines	14 (88 %)	9 (60 %)	0.11	7 (50 %)	10 (48 %)	0.95
Taking blood pressure medicines	5 (31 %)	11 (73 %)	0.04	3 (21 %)	8 (38 %)	0.26
Number of medications for control of glycemia, dyslipidemia, and blood pressure	3.9 (1.6)	4.6 (2.0)	0.54	1.1 (1.0)	1.9 (1.6)	0.08

*US* United States, *BMI* body mass index, *SBP* systolic blood pressure, *DBP* diastolic blood pressure, *HbA1c* glycated hemoglobin, *LDL cholesterol* low density lipoprotein cholesterol, *HDL cholesterol* high density lipoprotein cholesterol

At 24 months, no participant in either of populations of the IMM group achieved partial or complete remission. In contrast, in the RYGB group, the proportion of complete remission was 29 % and 14 % in the Taiwanese and American populations, respectively (*P* = 0.39). Overall, the proportion of complete or partial remission in Taiwanese and American participants was 57 % and 27 %, respectively (*P* = 0.08) (as Table 3).

**Table 3:** Remission rates at 24 months in RYGB patients

	Complete remission	Partial remission	Complete or partial remission
US (*N* = 22)	3 (14 %)	3 (14 %)	6 (27 %)
Taiwan (*N* = 14)	4 (29 %)	4 (29 %)	8 (57 %)
*P* value	0.39	0.39	0.08

*US* United States, *RYGB* Roux-en-Y gastric bypass

A univariate analysis showed that lower fasting glucose, higher stimulated C-peptide, and non-insulin use before RYGB predict complete or partial remission at 24 months in the RYGB patients. A multivariate analysis of predictors with *P* value <0.10 (except for fasting C-peptide, because it is highly correlated with stimulated C-peptide) showed that stimulated C-peptide is a significant predictor of DM remission (OR 2.22, *P* = 0.02) (as Table 4).

**Table 4:** Predictors of complete or partial remission at 24 months in RYGB patients

	OR (95 % CI)	*P* value
Univariate analysis*		
Nationality (Taiwan vs. US)	3.56 (0.86, 14.63)	0.08
Fasting glucose	0.984 (0.970, 0.999)	0.037
Fasting C-peptide	1.86 (0.92, 3.75)	0.084
Stimulated C-peptide	2.47 (1.23, 4.94)	0.011
On insulin	0.21 (0.05, 0.88)	0.033
Multivariate analysis^#^		
Model 1 AIC = 41.170		
Nationality (Taiwan vs. US)	2.04 (0.32,13.09)	0.45
Fasting glucose	0.99 (0.97, 1.005)	0.16
Stimulated C-peptide	2.17 (1.02, 4.58)	0.04
On insulin	0.84 (0.12,6.04)	0.86
Model 2: AIC = 39.202		
Taiwan	2.16 (0.38,12.38)	0.39
Fasting glucose	0.99 (0.97, 1.004)	0.16
Stimulated C-peptide	2.22 (1.11, 4.47)	0.025
Model 3: AIC = 37.954		
Fasting glucose	0.99 (0.97, 1.004)	0.15
Stimulated C-peptide	2.22 (1.13, 4.34)	0.02
Model 4: AIC = 39.148		
Stimulated C-peptide	2.47 (1.23, 4.94)	0.011

*OR* odd ratio, *US* United States, *RYGB* Roux-en-Y gastric bypass, *AIC* Akaike information criterion

*The following variables has *P* values of >0.10: age, sex, years since diagnosis of T2DM, BMI, waist, weight change from baseline to 24 months, HbA1c, SBP, DBP, LDL-C, HDL-C, triglycerides, total cholesterol, on non-insulin antidiabetic meds, on statins, on non-statin lipid-lowering meds, on BP meds, number of meds for control of glycemia, dyslipidemia, and BP

^#^Predictors in univariate analysis with *P* value <0.10 (except for fasting C-peptide since it is highly correlated with stimulated C-peptide) were included. Smaller AIC values are better.

## Discussion

In this report, we found that baseline BMI and waist circumference in Taiwanese participants were significantly less than in American participants. Taiwanese participants were slightly younger than the American cohort. In Asia, T2DM is characterized by onset at a relatively low BMI and a younger age [2]. Fewer Taiwanese participants were taking insulin and blood pressure medicines than American participants at the baseline of the DSS study. Nevertheless, uncontrolled T2DM remains a common problem in Taiwan [7].

In the first year of the DSS study, 47 % of the observed participants randomized to Roux-en-Y gastric bypass (RYGB) reached the triple endpoint goal compared with only 19 % of the participants randomized to treatment with (IMM) [13]. The proportion of participants achieving the triple endpoint goal decreased to 43 % and 14 % ,respectively, at 2 years with only 25 % of the gastric bypass group maintaining a complete remission of T2DM, and 42 % achieving partial remission [14].

In the 2-year report of the DSS, 36 % Taiwanese participants and 52 % American participants in the RYGB group achieved the triple endpoints, while only 19 % Taiwanese participants and 7 % American participants in IMM group achieved the triple endpoints; 86 % Taiwanese participants and 67 % American participants in RYGB group achieved the HbA1c target (<7.0 %) (53 mmol/mol), while only 19 % Taiwanese participants and 20 % American participants in IMM group achieved the target. No participant in either population in the IMM group met the definition of partial or complete remission of T2DM. Only 27 % of American and 57 % Taiwanese participants of the RYGB group achieved complete or partial remission of T2DM, less than reported in other trials. Lee and colleagues reported a randomized control trial to compare RYGB vs. sleeve gastrectomy for T2DM [11]. Remission of T2DM, defined as HbA1c <6.5 % (48 mmol/mol), was achieved by 28 (93 %) in the RYGB group and 14 (47 %) in the sleeve gastrectomy group. The STAMPEDE trial found 46 % of the RYGB group to have a HbA1c < 6.5 % (48 mmol/mol) without medication at 3 years [18]. The Longitudinal Assessment of Bariatric Surgery (LABS) consortium, a multicenter observational cohort study at 10 US hospitals in six geographically diverse clinical centers, reported 67.5 % of participants with T2DM achieving partial remission [8]. A multi-institutional, international study enrolling five Asian countries reported 79.3 % of patients undergoing RYGB achieved remission of T2DM, it defined as HbA1c < 6.0 % (42 mmol/mol) [9]. Possible explanations for the lower rate of remission in our study include greater severity of disease with a mean HbA1c of 9.6 % (81 mmol/mol) and a greater duration of T2DM—8.9 years in our population, although other randomized trials with higher rates of remission have included participants with average durations of T2DM of 6.4 to 10.4 years. It is also possible that our definition of remission was more rigorous by requiring a sustained HbA1c below the target value without diabetes medication at all visits from 12 to 24 months.

In our current report, 27 % of American and 57 % Taiwanese participants in the RYGB group achieved complete or partial remission of T2DM 2 years post-surgery (*P* = 0.08). The difference is not statistically significance in univariate and multivariate analysis, which may be limited by small sample size. The pathophysiology of T2DM is quite different in Asian vs. Caucasian populations. β-cell dysfunction with delayed and decreased insulin secretion appears to play a key role in the progression of T2DM especially in Asian populations [19]. Lee and colleagues [20] reported that RYGB facilitates immediate improvement in the glucose metabolism of inadequately controlled, mildly obese patients with T2DM, and the benefit is sustained up to 2 years after RYGB. The benefit in control of T2DM after RYGB appears to be mediated by a decrease in insulin resistance, an increase in early insulin response, and the total insulin secretion to glucose load [20]. National or ethnic differences in the response of T2DM to RYGB appear to exist and need further evaluation.

Lee and colleagues reported that remission rates of T2DM after RYGB were positively correlated with preoperative C-peptide levels, suggesting that this biomarker may be used to assist in the selection of patients with obesity-related T2DM for bariatric surgery [21]. In this 2-year report of DSS, we confirm that stimulated C-peptide is also a predictor of diabetes remission. Major limitation of this study is relatively small sample size to compare these two populations.

## Conclusion

This is the first randomized, controlled trial enrolling both Taiwanese and Americans comparing RYGB to a lifestyle intervention control group. In patients with mild obesity and T2DM, adding RYGB to lifestyle and medical management was associated with a greater likelihood of remission of diabetes in both Taiwanese and American. The residual beta-cell function of the participants appears to be the major factor to predict the remission. National or ethnic differences in response of T2DM to RYGB are still not clear. Larger and longer trials will be needed to fully evaluate the role of bariatric surgery in different populations, including effects and side effects. While the long-term risks and benefits of bariatric surgery should be weighed, the generalizing short-term to medium-term effects of RYGB for T2DM on Western cohorts and Asian populations appears appropriate.

## Abbreviations

DSS: Diabetes surgery study
IMM: Intensive medical management
RYGB: Roux-en-Y gastric bypass
CI: Confidence interval
T2DM: Type 2 diabetes mellitus
